# Comparison of three neutralizing broths for environmental sampling of low levels of *Listeria monocytogenes* desiccated on stainless steel surfaces and exposed to quaternary ammonium compounds

**DOI:** 10.1186/s12866-020-02004-1

**Published:** 2020-11-02

**Authors:** Fengmin Li, Zhihan Xian, Hee Jin Kwon, Jiyoon Yoo, Laurel Burall, Stuart J. Chirtel, Thomas S. Hammack, Yi Chen

**Affiliations:** 1 Office of Regulatory Science, Center for Food Safety and Applied Nutrition, Food and Drug Administration, College Park, MD USA; 2 Office of Applied Research and Safety Assessment, Center for Food Safety and Applied Nutrition, Food and Drug Administration, College Park, MD USA; 3 Office of Analytical Outreach, Center for Food Safety and Applied Nutrition, Food and Drug Administration, College Park, MD USA

**Keywords:** *Listeria monocytogenes*, Stainless steel surface, Transport broth, Desiccation, Sanitizer

## Abstract

**Background:**

An effective environmental sampling method involves the use of a transport/neutralizing broth with the ability to neutralize sanitizer residues that are collected during sampling and to maintain viability of stressed *Listeria monocytogenes* (*Lm*) cells.

**Results:**

We applied *Lm* onto stainless steel surfaces and then subjected *Lm* to desiccation stress for 16–18 h at room temperature (RT, 21–24 °C). This was followed by the subsequent application of Whisper™ V, a quaternary ammonium compound (QAC)-based sanitizer, diluted to 400 ppm and 8000 ppm of active quat, for 6 h. We then sampled *Lm* with sponges pre-moistened in three transport broths, Dey/Engley (D/E) broth, Letheen broth and HiCap™ broth, to generate environmental samples that contained sanitizer residues and low levels of stressed *Lm*, which were subsequently analyzed by an enrichment-based method. This scheme conformed with validation guidelines of AOAC International by using 20 environmental test portions per broth that contained low levels of *Lm* such that not all test portions were positive (i.e., fractional positive). We showed that D/E broth, Letheen broth and HiCap™ broth performed similarly when no quat or 400 ppm of quat was applied to the *Lm* contaminating stainless steel surfaces. However, when 8000 ppm of quat was applied, Letheen broth did not effectively neutralize the QAC in the samples. These comparisons were performed on samples stored under three conditions after collection to replicate scenarios of sample transport, RT for 2 h, 4 °C for 24 h and 4 °C for 72 h. Comparisons under the three different scenarios generally reached the same conclusions. In addition, we further demonstrated that storing Letheen and HiCap™ broths at RT for two months before sampling did not reduce their capacity to neutralize sanitizers.

**Conclusions:**

We developed a scheme to evaluate the ability of transport broths to neutralize QAC sanitizers. The three transport broths performed similarly with a commonly used concentration of quat, but Letheen broth could not effectively neutralize a very high concentration of QAC. The performance of transport broths was not significantly affected under the assessed pre-sampling and post-sampling storage conditions.

## Background

*L. monocytogenes* (*Lm*) survives under a wide range of temperature, pH and salt conditions, and is able to cross the intestinal barrier, the blood-brain barrier, and the maternofetal barrier [1]. Because of these features, *Lm* frequently contaminates foods and causes disease, listeriosis, in humans. Recently, *Lm* has been implicated in major outbreaks and recalls associated with contaminated cantaloupe, cheese, stone fruit, apples, packaged salads, and frozen vegetables, among others [2–6]. Despite continuous advances in food safety practices, disease surveillance, control and prevention, foodborne bacterial infections, particularly those caused by *Lm*, remain a significant public health concern.

*Lm* and other *Listeria* spp. are frequently isolated from food processing environments [7, 8]. It has been observed that the *Listeria* spp. isolated from such environments are usually sub-lethally injured, due to desiccation, exposure to sanitizers, high acidity, high osmotic conditions, and other environmental stresses [9]. The injured *Lm* may not survive the transport and storage procedures prior to testing, increasing the risk of false negative test results in public health testing laboratories. Ineffective rinsing in food processing facilities could leave sanitizer residues on food processing environments [10], and the likelihood of a false negative *Lm* testing result may be increased when the *Lm* cells and industrial sanitizer residues are in the same sampling area and collected together by the sampling device, resulting in additional injury to, or eradication of, the viable *Listeria* cells. This is especially concerning for the certain areas in food processing facilities that may have been exposed to higher-than-recommended concentrations of sanitizers due to inevitable uneven distribution of sanitizers when dry-powdered sanitizers or foam-sprayed sanitizers [11, 12] are used, and/or when rinsing is ineffective. Therefore, potential false negative results could be avoided if the neutralizing broths used for sample collection and transport can neutralize sanitizer residues that may exceed the recommended concentrations and still maintain the viability of stressed *Lm* cells.

To address these problems, a variety of neutralizing broths have been developed and evaluated [13–17]. For example, Letheen broth, a nonproprietary transport broth, combines nutrients with lecithin and polysorbate, which can neutralize phenols, hexachlorophene, formalin, ethanol and quaternary ammonium compounds (QAC). In comparison, Dey/Engley (D/E) broth contains polysorbate, a higher concentration of lecithin than that in Letheen broth, and additional neutralizing agents such as sodium thioglycolate, sodium thiosulfate, and sodium bisulfite which can neutralize additional antimicrobial and disinfectant chemicals, such as mercurial, iodine, chlorine, formaldehyde, and glutaraldehyde. HiCap™ Neutralizing Broth (HiCap) is a recently developed proprietary neutralizing broth [18] and claims to have the capacity to neutralize a variety of sanitizers. Other neutralizing broths, such as buffered peptone water (BPW), MCC buffer, and Neutralizing Buffer (NB) have been shown to be less effective than D/E and Letheen broths [19].

Additional factors that may influence the choice of transport broth include sample stability and storage convenience. For example, D/E broth is only stable at refrigerated temperatures before being used for sampling [20], which makes it less convenient for field inspection personnel. Stability at room temperature (RT) offers advantages such as storage convenience and space saving for cold storage. Furthermore, there are concerns that some neutralizing broth components such as casein and lecithin may leave residues in manufacturing facilities that could pose an allergen hazard if they enter the food supply [21]. Additionally, dairy-based casein products may not be used in meat processing facilities that follow Kosher practices and pork-based peptone is a problem for manufacturers of Halal or Kosher foods [21]. Another important consideration when testing neutralizing broths is that the ingredients in the broth do not interfere with downstream analyses for bacterial pathogens. For example, sodium thiosulfate and sodium bisulfite in some neutralizing broths may interfere with 3M™ Petrifilm™ [22, 23].

In order to properly assess the efficacy of any testing method, it is imperative that we generate samples that best replicate real world samples. This is not difficult to achieve for many types of food samples, since 1) foods can be purchased from grocery stores before microbial inoculation, 2) replicates and controls can be easily prepared, and 3) foods can be aged to allow inoculated bacterial cells to adapt to the food storage conditions before pathogen analysis. Thus, these artificially inoculated samples provide a good alternative to naturally contaminated samples. However, it is much more difficult to simulate the stresses that *Lm* in environmental samples undergo since the stressors are so diverse (desiccation, chemicals, acid, and salt). For example, if *Lm* is artificially inoculated directly onto sponges, it may not be subjected to the stresses that it is normally exposed to in food processing environments. To fully evaluate a detection method, it is preferable to replicate worst case scenarios and employ samples with low levels of highly stressed cells. The Microbial Method Validation Guideline of AOAC International [24] prescribes a scheme to partially achieve this purpose. Bacterial cells are inoculated onto environmental surfaces, desiccation stress is then induced by drying for 16–24 h, and the bacterial cells are collected using swabs/sponges. The level of cells after drying is very low, similar to the limit of detection for at least one of the compared methods and therefore the test portions may not all yield positive results with that method (i.e., fractional positive) [24]. Thus, to allow meaningful statistical comparison between methods, at least 20 test portions, in contrast to commonly used 3 or 5 replicates, inoculated at this low level are prepared and analyzed using each method, and the numbers of positive test portions are used for comparison. AOAC-specified analysis requires that the fractional positive rate is between 25% and 75% out of 20 test portions [24], and should be observed with at least one of the comparatively evaluated methods. This guideline is also harmonized with newly published ISO method validation standards [25].

We previously followed the AOAC guidelines to simulate environmental sampling, and test results from environmental samples containing *Lm* showed that two commonly used *Listeria* enrichment schemes did not effectively recover and enrich low levels of *Listeria* subjected to desiccation stress [26]. We demonstrated the critical importance of 48 h of culture enrichment for successful resuscitation [26]. Other factors of environmental testing, such as sampling device materials, storage conditions, and time between sample collection and laboratory analysis, were also evaluated in that study [26]. However, only D/E neutralizing broth, the one specified in U.S. Food and Drug Administration (FDA)‘s *Bacteriological Analytical Manual* (BAM) [17] and U.S. Department of Agriculture’s Microbiology Laboratory Guidebook (MLG) [27], was examined, and that study did not evaluate the effect of sanitizers on environmental testing [26]. The environmental sample preparation protocol by AOAC does not involve sanitizer as a factor, and previous studies following AOAC validation guidelines did not evaluate the effect of sanitizers. Thus, the primary objective of the present study was to develop a scheme to evaluate the efficacy of transport/neutralizing broths. We used this scheme to compare two neutralizing broths (Letheen and HiCap) to D/E broth for their ability to maintain the viability of desiccation- and sanitizer-stressed *Lm*, and their ability to neutralize relatively high concentrations of sanitizer residues. We followed AOAC Microbial Validation Guidelines for the number of test portions and data analysis [24].

## Results and discussion

**Fig. 1:**
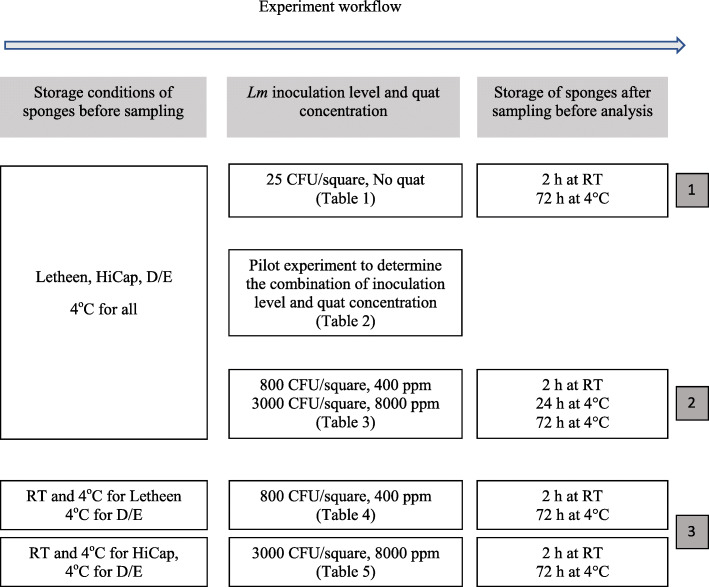
Three sets of experiments were performed in this study. The first set compared the three neutralizing broths without using Whisper™ V, a quaternary ammonium compound (QAC)-based sanitizer. The second set compared the three neutralizing broths by exposing inoculated *Lm* to QAC. Two quat concentrations were used, 400 ppm and 8000 ppm to represent a common concentration and a very high concentration. A pilot experiment was performed before this one to determine the appropriate combinations of *Lm* inoculum level and quat concentration that could yield fractional positive results in an AOAC validation scheme. After sponge sampling, three storage conditions were used to mimic same day sampling and enrichment-based analysis (i.e., 2 h at RT), enrichment-based analysis after overnight delivery of environmental samples (24 h at 4 °C) and enrichment-based analysis after over-the-weekend delivery of environmental samples (72 h at 4 °C). For the first two sets of experiments, three transport broths were stored at 4 °C before the experiments. The third set of experiments evaluated the stability of Letheen broth and HiCap broth stored at RT before sampling. Two separate evaluations were used because the inoculation levels and quat concentrations suitable to evaluate the two broths were different. The first one compared Letheen broth stored at RT and at 4 °C with D/E broth stored at 4 °C, and the second one compared HiCap broth stored at RT and at 4 °C with D/E broth stored at 4 °C. For the first and third set of experiments, we did not perform the analysis simulating the scenario of storing the sponge samples at 4 °C for 24 h due to logistical reasons

Transport broths typically combine nutrients with neutralizing agents to maintain the viability of bacterial cells during transport and to neutralize any sanitizer residues that may be surrounding the bacterial cells. Such sanitizer residues could cause false negative results in environmental testing. A variety of neutralizing agents were used in transport broths, such as lecithin, polysorbate, sodium thiosulfate, sodium bisulfite, and aryl sulphonate complex [14, 19–21]. Transport broths also typically contain agents that can neutralize pH. Previous studies have compared the neutralizing capacity of various buffer as potential transport media, even though their experimental designs were different from the current study [14, 19]. Park et al. evaluated several transport broths against three acidic sanitizers and one alkaline sanitizer by mixing transport broths with sanitizers before adding liquid Shiga toxin-producing *Escherichia coli* (STEC) cultures. Ward et al. mixed transport broths with different concentrations of sanitizers, including ~ 20 ppm to ~ 800 ppm of quat, before adding liquid *E. coli* into the mixture [21]. A similar approach was used by Sutton et al. [28], and the authors acknowledged that this study design provided no information on the recovery of sublethally injured bacteria due to exposure to sanitizers. Zhu et al. mixed *Lm* cells with 1 ppm of Quorum, a QAC-based sanitizer, before applying the mixture onto sponges or stainless steel surfaces; the authors also rinsed *Lm* biofilms containing high levels of *Lm* with 200 ppm of Quorum before sponge sampling. These studies showed that buffers that contain agents that can neutralize a variety of sanitizers were necessary for environmental sampling purposes. For example, PBS was shown to be effective to neutralize the effect of acetic acid against *E. coli* O157:H7 [29] due to pH neutralization, however, it did not contain any components that can neutralize commercial sanitizers, and was proved experimentally to be ineffective for neutralizing commercial sanitizers [14]. In our study, we developed a scheme to comparatively evaluate neutralizing broths. This scheme induced stress to *Lm* by drying cells and then exposing cells to sanitizers, and we chose three transport broths that were previously shown to have relatively high capacity for comparison. The overall experiment design is explained in Fig. 1.

### Comparison of Letheen broth, D/E broth and HiCap broth for collecting cells subjected to desiccation stress only

When environmental samples are collected, swab or sponge samplers are generally pre-moistened in transport broths to increase the sampling efficacy [17, 30]. After sampling, the pre-moistened swabs or sponges are placed back into the sample bag containing leftover transport broths. When the sampling areas are very wet, dry samplers are used for sampling and then returned to a sample bag containing transport broths.

In this study, *Lm* and sanitizers were both dried before sampling, and thus, we used the sponges (approximately 4 cm long × 4 cm wide × 1.5 cm thick) pre-moistened in 10 mL transport broth. These sponges absorbed all 10 mL with no running liquid in the sample bag. One sponge was used to sample one sampling section (10 cm × 10 cm square), resulting an environmental test portion. Two-way comparisons between the Letheen broth and D/E broth and between the HiCap broth and D/E broth (Table 1) showed that all three broths were similar in their capability to maintain the viability of *Lm* cells subjected to desiccation stress. We reached the same conclusions using two scenarios of sample storage after sample collection and before qualitative analysis: 1) the test portions stored at RT for 2 h simulating same day transport of environmental samples to the laboratory and same day enrichment-based analysis; 2) the test portions stored at 4 °C for 72 h simulating enrichment-based analysis after over-the-weekend delivery of environmental samples. Bacteria populations could reduce in transport broths during storage at 4 °C [20], and that is why we evaluated different storage durations after sponge sampling. In this preliminary experiment without applying sanitizers, we did not perform the analysis simulating the scenario of storing the sponge samples at 4 °C for 24 h due to logistical reasons.

**Table 1:** Number of positive test portions out of 20 test portions generated with different neutralizing broths when the initial inoculation level was ~ 25 CFU/square and no Whisper**™** V was applied to *Lm* cells before sampling. Neutralizing broths were stored at 4 °C before use for the sample collection

Sample storage condition between collection and analysis	Number of positive test portions out of 20 test portions collected using each neutralizing broth		
	D/E	Letheen	HiCap
2 h at RT (*p* = 0.51)^a^	11	8	7
72 h at 4 °C (*p* = 0.48)	5	9	6

^a^Extended Fisher’s exact test was performed for each row among different enrichment broths

We followed Microbiological Method Validation Guidelines of AOAC International [24] by desiccating *Lm* cells on stainless steel surfaces for 16–18 h. This protocol was also referred to in the recently published ISO method validation standards (ISO 16140-2) [25]. This approach allows relatively straightforward replication and has been used to validate a variety of methods for environmental sampling of foodborne pathogens [31–33]. Even though this desiccation process was not sufficient for *Lm* to form mature biofilms [34], it induced desiccation of *Lm* on stainless steel surfaces that could be used to evaluate the efficacy of enrichment schemes to resuscitate stressed *Lm* [26]. Specifically, we found that *Lm* subjected to such desiccation stress could not fully recover and grow to detectable levels after 24 h of enrichment in several commonly used *Listeria* enrichment broths [26]. Another study also showed that samples containing *Lm* that were desiccated on stainless steel plates for 1 h before sampling yielded fewer positive results than samples collected from plates with no cell drying when the same 24 h culture enrichment was used for pathogen analysis [35]. Therefore, in order to evaluate the performance of a qualitative analytical method, we may not necessarily need mature biofilms. Nonetheless, in future studies, we could explore the option of using mature biofilms to evaluate various elements of detection methods (e.g., sampling devices).

### Determination of the Whisper™ V concentration and inoculation level that could challenge neutralizing broths while yielding fractional positive results needed for method comparison

Since the AOAC-specified protocol to compare environmental testing methods does not involve sanitizers, we explored different schemes of adding sanitizers in our preliminary work. The goal was to add sanitizers at concentrations similar to or higher than those commonly used in food processing environments, while still keeping the levels of *Lm* relatively low. We first added sanitizers directly to swabs/sponges pre-moistened with transport broths and then used the pre-moistened sponges to sample *Lm* inoculated on stainless steel surfaces. Similar to previous approaches that mixed sanitizers with transport broths before adding bacteria cells [14, 21], this approach could be used to evaluate the ability of transport broths to neutralize sanitizers; however, the sanitizers might be fully or partially neutralized before the sponges were used to collect *Lm*, and any remaining sanitizers may not be enough to induce sufficient stress of the *Lm* cells. We also mixed fresh cultures of *Lm* with sanitizers before spreading them onto stainless steel surfaces, but *Lm* cells did not survive in sanitizer concentrations above 50 ppm of quat (unpublished data). We also applied sanitizers to stainless steel surfaces first and then added *Lm* cells onto the surfaces, however, *Lm* cells either died off very fast on the surfaces or very high levels of *Lm* were needed (unpublished data). These preliminary works led to the development of our current scheme. Even though our primary focus was to evaluate the sanitizer-neutralizing capacity of transport broths, we would still like to test whether these transport broths can maintain the viability of stressed *Lm*. Thus, we first induced desiccation stress by drying *Lm* for 16–18 h, and then applied sanitizer over *Lm* and dried for another 6 h. This way the total time of *Lm* drying on stainless steel surfaces was still within the range of 16–24 h as prescribed by the AOAC guidelines [24]. The current scheme also replicated real world conditions where surfaces are first contaminated and then sanitized. Waiting for any liquid (i.e., cultures and sanitizers) to dry also ensured the sampling efficacy of pre-moistened sponge samplers because these samplers were nearly saturated with transport broths.

We performed a pilot study to test the combination of inoculation levels and Whisper™ V concentrations that could be used to compare the neutralizing broths and to determine the appropriate combination needed to obtain fractional positive test results in a full-scale AOAC validation. The pilot test portions were analyzed on the same day of collection. The results with ~ 800 CFU/square (Table 2) of *Lm* indicated that fractional positive results among 20 test portions might be achieved when Whisper™ V concentrations of 400 ppm and 800 ppm of quat were applied to *Lm* cells before sampling. The results with ~ 3000 CFU/square (Table 2) inoculum indicated that fractional positive results could be achieved when Whisper™ V concentrations of 1600 ppm, 4000 ppm and 8000 ppm of quat were applied. The manufacturer-recommended concentrations for Whisper™ V was 150 to 1200 ppm of active quat for different facility areas and sanitizing systems with 200–800 ppm among the most common concentrations. According to another guidance document, the recommended use levels for quat ranged from 200 ppm for equipment, 800 ppm for floors and drains, 1800 ppm for floor mats, 2400 ppm for foot baths to 2000 or even 5000 ppm for mold treatment of walls and ceilings [36]. Application of foam-based QAC or dry QAC crystals is used in certain food processing environments, sometimes with no rinsing afterwards [11, 12]. A pre-moistened sponge absorbing dry QAC crystals remained on environmental surfaces or compositing/pooling sponges collected from multiple locations could result in very high concentrations of active quat in the collected environmental samples. Thus, we first chose ~ 800 CFU/square and 400 ppm of quat to compare transport broths for the full-scale AOAC validation, and we further chose ~ 3000 CFU/square and 8000 ppm of quat as another combination. Thus, our scheme allowed the evaluation of transport broths using low levels of *Lm* exposed to a QAC sanitizer at concentrations similar to and higher than those commonly used in food processing environments.

**Table 2:** Number of positive test portions out of five test portions generated with D/E, Letheen and HiCap neutralizing broths when using an initial inoculation level of ~ 800 CFU/square and ~ 3000 CFU/square of *Lm* and different concentrations of Whisper**™** V were applied to *Lm* cells before sampling. Neutralizing broths were stored at 4 °C before sample collection

Inoculation levels	Neutralizing Broth	Number of positive test portions at each quat concentration				
		8000 ppm	4000 ppm	1600 ppm	800 ppm	400 ppm
~ 800 CFU/square	Letheen	0	3	1	2	4
	D/E	1	0	1	3	3
	HiCap	1	1	1	3	4
~ 3000 CFU/square	Letheen	0	4	4	5	5
	D/E	5	3	2	4	5
	HiCap	3	5	4	5	5

### Comparison of Letheen broth, D/E broth and HiCap broth for collecting cells subjected to desiccation stress and then sanitizer stress

With the inoculation level of 800 CFU/square and 400 ppm of quat, the number of positive test portions collected using sponges pre-moistened with D/E, Letheen and HiCap neutralizing broths were not statistically different when test portions were analyzed on the same day of sample collection (*p* = 0.44, Table 3) and when test portions were stored at 4 °C for 72 h (*p* = 0.15, Table 3). When test portions were stored at 4 °C for 24 h, differences in the recovery of stressed *Lm* with neutralizing broths resulted in a *p* value of 0.06. Since the Fisher’s exact test is conservative, we performed pairwise comparisons when *p* value was 0.06, which showed that the number of positive test portions collected with sponges pre-moistened with D/E broth was lower than those with Letheen broth using enrichment-based analyses (*p* = 0.04), but no differences were observed between the D/E and HiCap broths (*p* = 0.13). The same conclusions were obtained when the extended Fisher’s exact and chi-square tests were performed to compare D/E broth and Letheen broth or D/E broth and HiCap broth. When combining all scenarios, our data indicated that all three neutralizing broths had similar abilities to neutralize Whisper™ V and to maintain the viability of desiccation- and sanitizer-stressed *Lm* cells when the sanitizer contained 400 ppm of quat.

**Table 3:** Number of positive test portions out of 20 test portions generated for D/E, Letheen, and HiCap neutralizing broths using an inoculation level of ~ 800 CFU/square *Lm* followed by an application of 400 ppm of quat and an inoculation level of ~ 3000 CFU/square *Lm* followed by an application of 8000 ppm of quat before sampling. Neutralizing broths were stored at 4 °C before use

Initial inoculation level and quat concentration	Storage condition between collection and analysis	Number of positive test portions collected using each broth^a^		
		D/E	Letheen	HiCap
~ 800 CFU/square of *Lm* and 400 ppm of quat	2 h at RT (*p* = 0.44)	18	15	15
	24 h at 4 °C (*p* = 0.06)	13 a	19 b	18 ab
	72 h at 4 °C (*p* = 0.15)	14	19	16
~ 3000 CFU/square of *Lm* and 8000 ppm of quat	2 h at RT (*p* = 0.0002)	10 a	1 b*	13 a
	24 h at 4 °C (*p* = 0.002)	12 a	2 b*	11 a
	72 h at 4 °C (*p* = 0.0008)	11 a	2 b*	13 a

^a^Extended Fisher’s exact tests for each row among different enrichment broths, and *p* values are listed in the parenthesis after each storage condition. When p was less than 0.1, pairwise comparisons were performed; results sharing the same letter were not statistically different (*p* > 0.05) and * indicates *p* < 0.01 between different letters. *p* values of pairwise comparisons are not listed

When the inoculation level was ~ 3000 CFU/square and the QAC contained 8000 ppm of quat, D/E and HiCap broths generated statistically equivalent results (*p* > 0.5), but Letheen broth generated a statistically smaller number of positive test results than D/E and HiCap broths (*p* < 0.006 for all pairwise comparisons). Thus, the results showed that Letheen broth did not effectively neutralize high (8000 ppm of quat) concentrations of Whisper™ V residue picked up by the sponge samplers (Table 3). We reached the same conclusions when simulating: 1) same day transport of collected environmental samples and same day enrichment-based analysis; 2) enrichment-based analysis after overnight delivery of collected environmental samples in cold storage; and 3) enrichment-based analysis after over-the-weekend delivery of collected environmental samples in cold storage. The multiple comparisons by extended Fisher’s exact test generated the same conclusions as the chi-square tests performed between D/E broth and Letheen broth and between D/E broth and HiCap broth. In two other studies that used different experiment designs, under certain conditions D/E broth was also shown to be more effective than Letheen broth in sanitizer neutralization [19, 21].

In order to better understand the effect of desiccation and sanitizer on inoculated *Lm* cells, we quantified the culturable *Lm* cells on stainless steel surfaces. We found that when 800 CFU/square of *Lm* (i.e., 2.9 log CFU/square) was inoculated and dried for 16–18 h, culturable *Lm* remaining on 20 stainless steel squares varied from 2.2 to 2.5 log CFU/square with an average of 2.3 log CFU/square. Therefore, the culturability loss was 0.6 log CFU/square. This is consistent with previous analyses showing that inoculating at that high level without subsequent exposure to sanitizer would yield 100% positive results among 20 test portions [26]. When 3000 CFU/square of *Lm* (i.e., 3.5 log CFU/square) was inoculated and dried, culturable *Lm* remaining on stainless steel surfaces varied from 2.6 to 3.0 log CFU/square with an average of 2.7 log CFU/square. Therefore, the culturability loss was 0.8 log CFU/square, similar to that with inoculum of 800 CFU/square. When 800 CFU/square was inoculated, dried and then exposed to 400 ppm of quat for 6 h, culturable *Lm* were observed on 15 out of 20 stainless steel squares and their levels varied from 0.5 to 1.5 log CFU/square (i.e., 3 to 32 CFU/square) with an average of 1.0 log CFU/square (i.e., 10 CFU/square). Therefore, the sanitizer application onto desiccation-stressed *Lm* further reduced the culturability of *Lm* by 1.3 log CFU/square. The *Lm* rinsed off the stainless steel surfaces were subjected to direct plating enumeration, not enrichment-based analysis, although it is not unreasonable to speculate that any rinsate containing culturable *Lm* would have generated positive result after 48 h enrichment. Our approach to recover culturable *Lm* cells appeared to have generated results consistent with sponge sampling and subsequent enrichment-based analysis. The low levels of remaining *Lm* could explain why we obtained fractional positive results from 20 test portions. Out of the 15 sampling squares that contained *Lm* culturable on brain heart infusion (BHI) agar, 10 yielded colonies on Rapid’*L.mono* agar. The ratio of culturable *Lm* on Rapid’*L.mono* agar to those on BHI agar on the 15 sampling squares varied from 0 to 1, with a median of 30%, indicating that on average 70% of culturable *Lm* cells in a given square could not grow on Rapid’*L.mono* after 48 h of incubation. This result should be interpreted with caution since very low levels of culturable *Lm* remained after desiccation and sanitizer exposure, and there were only 0 to 4 colonies on each agar plate, affecting the accuracy and precision of direct plating enumeration. Nonetheless, the different counts on BHI agar and Rapid’*L.mono* agar indicated that *Lm* were stressed. We did not quantify *Lm* after inoculation at 3000 CFU/square and exposure to 8000 ppm of quat due to much larger volume of transport broths needed to neutralize QAC residue, however, since the final results after enrichment-based analysis were also in the fractional positive range, the levels of *Lm* prior to enrichment were likely similar between the two inocula/quat combinations.

In this study, we added exposure of low levels of *Lm* to sanitizer as an extra step after the AOAC-prescribed desiccation only treatment, and the sanitizer concentrations were similar to or higher than those used in food processing environments. Our comparison showed that when the quat concentration in the environmental sampling areas was similar to the commonly recommended concentration, all three transport broths had a similar capacity to neutralize QAC. However, when the amount quat in the environmental sampling areas was very high, Letheen broth was not as effective as D/E broth and HiCap broth, although this likely only represents a very small fraction of real-world scenarios. We used Whisper™ V and selected two combinations of sanitizer concentration and *Lm* inoculation level in this study, but other sanitizers and concentrations should be used to evaluate an appropriate transport broth for a specific food processing facility. Different areas at different production shifts of the facility can be sampled and sanitizer residue concentrations determined prior to choosing the best sanitizer and concentration to evaluate transport broths. In a study aimed at evaluating *Lm* resistance to sanitizers, *Lm* biofilm formation on stainless steel plates led to enhanced resistance to quat [37]. Therefore, if we allowed *Lm* to form mature biofilms, we could have applied even higher concentrations of QAC or inoculated lower levels of *Lm* for the AOAC-type validation. Nonetheless, the QAC concentrations used in our study were similar to or higher than those recommended for food industry use, and *Lm* levels after desiccation stress and sanitizer stress were relatively low (i.e., 3 to 32 CFU/square when the initial inoculum was 800 CFU/square). It is intriguing to find out whether drying for 16–24 h used in our study could have enhanced the resistance of *Lm* to QAC. Transcriptomic analysis of *Lm* desiccated on stainless steel showed that genes involved in response to other stresses were also upregulated [38]. Desiccation-treated *Salmonella* showed enhanced tolerance to multiple disinfectants, such as QAC, ethanol, sodium hypochlorite, hydrogen peroxide, and bile salts [39]. Therefore, we could not exclude the possibility that desiccated *Lm* cells, without forming mature biofilms, could have enhanced resistance to QAC.

Our data also raised concerns on compositing/pooling sponges from different sampling areas for analysis, because this could increase the chance of pooling higher-than-recommended concentrations of sanitizer residues in one area together with *Lm* from other areas that did not contain sanitizer residues. As a result, the *Lm*-containing areas that could have yielded individual positive samples might not have been identified. Therefore, prior knowledge of the distribution of sanitizer residues in a food processing facility is very critical in designing an effective environmental sampling and testing strategy.

In this study, sampling efficacy should not affect our comparisons since we followed the same sponging practices among different test portions and treatments. Nonetheless, effective sampling techniques would help maintain consistency in sponge sampling. To evaluate sampling efficacy, we rinsed off 20 stainless steel sampling squares after inoculation, desiccation, exposure to QAC, and sponge sampling, and could not recover any culturable *Lm* in the rinsates (limit of detection, 1.25 CFU/square), and this was true with either 800 CFU/square of inocula and 400 ppm of quat or 3000 CFU/square of inocula and 8000 ppm of quat. This indicated that our sampling technique was satisfactory and yielded consistent results. The inocula and QAC were visually dry before sampling, which helped ensure the efficacy of sampling by pre-moistened sponges. The use of smooth stainless steel surfaces also helped effective sampling. We used sponges made of polyurethane in this study and our previous study using the same inoculation and desiccation approaches had showed that cotton, cellulose, polyester, and polyurethane materials were equivalent for picking up *Lm* from stainless steel surfaces [26]. Our findings were consistent with recent studies that did not observe significant effect of sponge/swab materials on recovery of *Lm* from various surfaces [35, 40].

After enrichment-based analysis, all positive samples contained at least 10^4^ CFU/ml of *Lm* and thus we only counted the number of positive samples for comparison. We had attempted to quantify the number of *Lm* cells collected by each sponge sampler before enrichment, however, because sponges contained very low levels of *Lm* and homogenizing using a stomacher could not fully release the *Lm* cells from sponges to liquid for subsequent enumeration, we could not obtain accurate enumeration (unpublished data). Accurate enumeration may have been possible if very high concentrations of *Lm* cells (e.g., > 3 log CFU) were collected on the sponges; however, this would not result in fractional positive results after enrichment-based analysis or allow AOAC/ISO-style comparative evaluations. Nonetheless, quantitative analysis of *Lm* cells on the sponges after sampling could provide an alternative evaluation of the efficacy of environmental sampling, although it would be a very different experimental design that can be investigated in future studies. Another approach to evaluate these transport broths for environmental sample collection would be to obtain authentic samples from food manufacturing facilities. However, it is challenging to obtain multiple nearly identical sets of positive samples for well controlled method comparisons. Another alternative approach would be to collect a very large number of samples from diverse areas, where the number of samples would allow an assessment across the variable samples. However, this would be a relatively large study that presents logistical challenges, both in obtaining a large and diverse sample set and in processing these samples.

### Evaluation of Letheen broth and HiCap broth stored at RT before use by comparison to those stored at 4 °C

Both Letheen broth and HiCap broth were claimed to be stable at RT before being used for environmental sampling [21]. Therefore, we performed experiments to evaluate these claims. We compared these broths after storage at RT and at 4 °C before being used for sample collection, and we also included D/E broth stored at 4 °C for each comparison. We used 400 ppm of quat when Letheen broth was evaluated and 8000 ppm of quat when HiCap broth was evaluated because earlier data showed that these were the concentrations that would yield fractional positive results for each of these broths. Letheen broths stored at RT and at 4 °C for 2 months enabled the recovery of statistically equivalent numbers of positive test portions, as did the HiCap broth. Both broths stored at RT also generated equivalent results to D/E broth stored at 4 °C (Tables 4 and 5). We reached the same conclusions when simulating: 1) same day analysis of samples; 2) analysis after overnight delivery of samples in cold storage; and 3) analysis after over-the-weekend delivery of samples in cold storage. While we evaluated the storage at RT and at 4 °C for sponges before sampling, we did not evaluate storage of sponges after sampling for more than 24 h at RT because previous studies suggested that bacteria could grow in transport broths at RT due to the nutrients present in transport broths and this growth was not selective, and thus background flora could outgrow *Lm*, negatively affecting subsequent selective enrichments [20].

**Table 4:** Number of positive test portions out of 20 test portions generated with Letheen broth stored at RT, Letheen broth and D/E broth stored at 4 °C before sample collection when the initial inoculation level was ~ 800 CFU/square and 400 ppm of Whisper™ V was applied to *Lm* cells before sampling

Storage condition between sample collection and analysis	Number of positive test portions collected using neutralizing broths stored under different conditions before use^a^		
	Letheen at RT	Letheen at 4 °C	D/E at 4 °C
2 h at RT (*p* = 1)^a^	15	16	15
72 h at 4 °C (*p* = 0.25)	16	17	12

^a^Extended Fisher’s exact tests for each row among different enrichment broths

**Table 5:** Number of positive test portions out of 20 test portions generated with HiCap broth stored at RT, HiCap broth and D/E broth stored at 4 °C before sample collection when the initial inoculation level was ~ 3000 CFU/square and 8000 ppm of Whisper™ V was applied to *Lm* cells before sampling

Storage condition between sample collection and analysis	Number of positive test portions collected using neutralizing broths stored under different conditions before use^a^		
	HiCap at RT	HiCap at 4 °C	D/E at 4 °C
2 h at RT (*p* = 0.94)^a^	11	12	13
72 h at 4 °C (*p* = 0.71)	14	13	11

^a^Extended Fisher’s exact tests for each row among different enrichment broths

In our study, we designed a scheme to evaluate transport broths by using desiccation- and sanitizer-treated *Lm*. We performed evaluation using one sanitizer representing QAC. Due to the high number of test portions, controls for each broth, and limited biosafety cabinet space, we compared three broths side by side and used two sanitizer concentrations. Real-world environmental sampling and testing can be very complex. There are many other types of sanitizers in addition to QAC, and there are many different QACs. Different food processing environments can have very different distributions of sanitizer residues at different times of production. There are additional commercially available transport broths. Therefore, the primary objective of this study was not to offer definitive guidance on the three neutralizing broths; rather, we developed a scheme that can be adapted later to evaluate various elements of environmental testing methods. The core principles of this scheme were to induce desiccation stress and sanitizer stress to low levels of *Lm* cells, to use the sanitizer concentrations similar to or higher than those found in different areas of a food processing facility, and to use relatively large number of test portions. In future studies, other neutralizing broths, sanitizers, sanitizer concentrations, and other strains of *Lm* or *Listeria* spp. can be incorporated into such a scheme. In our previous study aimed at evaluating enrichment schemes, competing microflora were co-inoculated with *Lm* at concentrations a log higher than *Lm,* and they significantly affected the performance of enrichment-based analyses [26]. Zhu et al. observed that the presence of background flora could affect how transport broths maintain the viability of *Lm* [19]. Specifically, certain transport broths appeared to better facilitate *Lm* to outcompete certain background flora [19]. Therefore, future work to evaluate transport broths should inoculate *Lm* along with background flora before exposure to sanitizers and subsequent sponge sampling and analysis. Background flora would possibly affect the *Lm* stress response during exposure to sanitizers, thereby affecting how transport broths maintain the viability of *Lm*. Furthermore, there has been no investigation on whether background flora could also interfere with the sanitizer-neutralizing capacity of transport broths. In addition, real-world environmental samples could also contain organic soils and/or food debris, which could very likely interfere with the performance of transport broths. Therefore, a comprehensive evaluation of any compounds that might interfere with transport broths from different food categories and different types of food processing environments is needed to further evaluate the efficacy of transport broths. Our preliminary work applying rinsates of raw produce along with *Lm* onto stainless steel surfaces before desiccation and sanitizing treatments did not reach different conclusions regarding the relative neutralizing capacity among Letheen, D/E and HiCap broths (unpublished data). In our previous study, we also experimented with different types of environmental surfaces (e.g., rubber, plastic, wood, or cast iron), which did not affect our conclusions on the relative performance of enrichment schemes. However, the potential effects of environmental surface types warrant further investigations. *Lm* likely endure different degrees of desiccation stress when drying on different surfaces, and this may affect subsequent sanitizer stress, sanitizer neutralization and viability in transport broths. In addition, we could allow *Lm* to form more mature biofilms before applying sanitizers. *Lm* biofilms may have enhanced resistance to sanitizers, and in order to obtain fractional positive results, we could reduce the levels of initial inocula or increase the QAC concentrations so that we further challenge the qualitative methods and potentially expose weaknesses of any method.

## Conclusions

In summary, we developed a scheme to apply *Lm* onto experimental surfaces, subject *Lm* to desiccation stress, and subsequently apply different concentrations of a QAC sanitizer onto desiccated *Lm*. This allowed us to generate environmental samples that contained sanitizer residues and low levels of stressed *Lm* after sponge sampling. We used this scheme to evaluate three neutralizing broths, D/E, Letheen and HiCap, and found that these broths provided similar results when no sanitizer or sanitizer at the industry-recommended concentrations was applied to *Lm* cells desiccated on stainless steel surfaces; however, the recovery of *Lm* from sponges pre-moistened and transported in Letheen broth was significantly lower than D/E or HiCap broths when a very high concentration of sanitizer was used. In general, whether collected samples were analyzed on the same day of sample collection or after storage at 4 °C for 72 h, there were no differences in the conclusions of broth comparisons. HiCap broth and Letheen broth are stable under RT, at least for the 2-month period evaluated in this study.

## Methods

### Environmental surfaces and bacterial strains

Food grade stainless steel plates and a single strain of *Lm* (HI-051, serotype 1/2a), isolated from an environmental source were used for all the experiments. The strain was cultured overnight in BHI with 150–200 rpm shaking at 37 °C to reach ~ 4 × 10^9^ CFU/ml.

### Preparation of environmental samples artificially inoculated with desiccation- and sanitizer-stressed *Lm*

We prepared environmental test portions by modifying a previously described method [26]. Briefly, large stainless steel plates were divided into 10 cm × 10 cm designated sampling squares. An overnight liquid culture of *Lm* was diluted to appropriate concentrations in BHI broth, and 320 μl of diluted culture was evenly spread onto each designated square on the stainless steel plates. We found that 320 μl of liquid is the minimum amount that would allow relatively even spread of the inoculum. The inoculated plates were left at RT (21–24 °C) and 50–60% relative humidity to dry for 16–18 h in biosafety cabinets with no air flow. Whisper™ V, a quaternary ammonium chloride sanitizer (active ingredients, 3% dimethyl benzyl ammonium chloride, 2.25% octyl decyl dimethyl ammonium chloride, 1.35% didecyl dimethyl ammonium chloride, 0.90% dioctyl dimethyl ammonium chloride. Ecolab Inc., St Paul, MN), was then added onto the treated stainless steel plates at 320 μL/square. Whisper™ V is a low-foaming, liquid sanitizer, which is usually used in both raw and ready-to-eat meat and poultry processing facilities. It also can be used for a variety of applications including sanitation of equipment, hard surfaces, and shell eggs intended for food. The recommended concentrations of Whisper™ V ranged from 150 to 400 ppm of quat for food processing equipment to 400–1200 ppm of quat for floors per manufacturer instructions. Whisper™ V application was followed by another 6 h of drying at RT. Each sampling square was subsequently sampled with a pre-moistened sponge, resulting in an environmental test portion. We first applied no Whisper™ V to induce only desiccation stress on *Lm* cells using the previously suggested inoculation level of 25 CFU/square of *Lm* [26] to reach fractional positive test results according to AOAC validation guidelines.

### Determination of the Whisper™ V concentrations and *Lm* inoculation levels that can be used to compare the efficacy of transport/neutralizing broths

Achieving fractional positive results required multiple experiments to determine the appropriate *Lm* inoculation levels, and thus we first conducted a pilot experiment to determine the quat concentrations that are suitable to compare neutralizing broths and the corresponding inoculation level that, after *Lm* stress, returned fractional positive test results. Five concentrations of Whisper™ V were evaluated: 8000 ppm, 4000 ppm, 1600 ppm, 800 ppm and 400 ppm of quat. We also evaluated two *Lm* inoculation levels (~ 800 CFU and ~ 3000 CFU per square) using five test portions per inoculation level. Once we determined the appropriate combinations of quat concentration and inoculation level, we proceeded with a full-scale experiment and prepared environmental test portions as described below.

### Sampling with sponges pre-moistened with different neutralizing broths, subjected to different storage conditions after sample collection and before enrichment-based qualitative analysis

The EZ Reach™ polyurethane sponge samplers pre-moistened with 10 mL Letheen broth (#EZ-10LET-PUR, World Bioproducts, LLC, WA), 10 mL D/E broth (#EZ-10DE-PUR, World Bioproducts, LLC, WA), or 10 mL HiCap Neutralizing Broth (#EZ-10HC-PUR, World Bioproducts, LLC, WA) were used to collect *Lm* cells from inoculated stainless steel surfaces. For sampling, even and firm pressure was applied to push the sampler in one direction across each sampling square: 10 times vertically, followed by 10 times horizontally, and then 10 times diagonally. After sampling, sponges containing sampled *Lm* were hand massaged in its neutralizing broth 10–20 times, and were subjected to one of three different storage conditions meant to replicate transport and delivery conditions: 1) test portions stored at RT for 2 h to simulate same day sample delivery and same day qualitative analysis; 2) test portions stored at 4 °C for 24 h to simulate overnight delivery of samples before qualitative analysis; and 3) test portions stored at 4 °C for 72 h to simulate analysis after over-the-weekend delivery of samples.

### Detection of *Lm* from environmental samples using enrichment-based analysis

After sample collection and storage, environmental test portions were analyzed according to FDA’s *Bacteriological Analytical Manual* (BAM). Briefly, each test portion (i.e., sponge containing *Lm* and moistened with 10 mL neutralizing broth) was mixed with 90 ml buffered *Listeria* enrichment broth (BLEB) (Oxoid, Thermo Fisher Scientific, Waltham, MA) for incubation at 30 °C. After initial 4 h of incubation, *Listeria* selective agents (Oxoid, Thermo Fisher Scientific, Waltham, MA) were added and incubation was continued at 30 °C for another 44 h. Enrichment cultures were then streaked onto Agar *Listeria* Ottavani & Agosti (ALOA) agar (bioMérieux, Inc., St Louis, MO) for incubation at 37 °C for 24 to 48 h. Blue colonies with a white opaque halo were presumptive positive for *Lm*, and subsequently confirmed using VITEK® MS (bioMérieux, Inc., St Louis, MO).

### Number of test portions and inoculation levels per method

The comparison of neutralizing broths was performed following AOAC Microbial Validation Guidelines [24]. For each neutralizing broth, 20 test portions were prepared. The inoculation level and physiological stress of *Lm* resulted in fractional positive (25% to 75% positive) results after enrichment-based detection of *Lm* for at least one broth in each comparison. This experimental design allowed for the comparison of two methods when the *Lm* levels in the test portions were close to the limit of detection of at least one method. Per requirement of AOAC validation guidelines, additional test portions were also prepared for each neutralizing broth: five test portions containing high levels (at least 10 times the level that would yield fractional positive results) and expected to yield 100% positive after enrichment-based analysis, and another five test portions containing no *Lm* (i.e., stainless steel surface inoculated with blank BHI) serving as negative controls.

### Evaluation of Letheen broth and HiCap broth stored at RT and at 4 °C before these two broths were used for sampling

Storage at RT was listed as an option for Letheen broth [41] and HiCap broth, and thus, their performance when stored at RT before being used for sampling was evaluated by comparison with these broths stored at 4 °C and with D/E broth stored at 4 °C. For the comparison involving HiCap broth, ~ 3000 CFU/square inoculum and 8000 ppm of quat were used. For the comparison involving Letheen broth, ~ 800 CFU/square inoculum and 400 ppm of quat were used. For each of the Letheen broth and HiCap broth, broths stored at RT for 2 months, the same broths stored at 4 °C for 2 months, and the D/E broth stored at 4 °C for 2 months were compared.

### Quantitation of *Lm* cells remaining after desiccation, after sanitizer exposure and after swabbing

In order to understand the reduction or loss of culturability of *Lm* after stress, we performed additional experiments to determine the level of remaining *Lm* in the 20 inoculated squares. To determine the levels of *Lm* after desiccation, we used the same inoculation levels, 800 CFU/square and 3000 CFU/square. To recover the remaining *Lm* after desiccation treatment, each sampling square was rinsed with 2 mL of sterile PBS by aspirating and dispensing at least 30 times using a micropipette moving across the square [42]. Around 1.6 mL of PBS was able to be collected and plated on 8 BHI agar plates (i.e., 200 μL/agar plate) for incubation and enumeration. A portion of representative colonies on BHI agar were streaked onto Rapid' *L.mono* (Bio-Rad Laboratories, Hercules, CA) chromogenic agar and subjected to API *Listeria* test (bioMerieux, Inc., St. Louis, MO) for confirmation as *Lm*. To determine the levels of *Lm* after desiccation and sanitizer exposure, we used the inoculation level of 800 CFU/square and 400 ppm of quat. We used the approach described above to recover *Lm* in each square but used D/E broth instead of PBS to rinse *Lm* cells exposed to and surrounded by sanitizers. After rinsing, we plated the 1.6 mL of collected D/E broth onto four BHI agar plates and four Rapid *L’mono* chromogenic agar plates. The counts on BHI agar were used to estimate the total levels of culturable *Lm* remaining on stainless steel surfaces and the counts on Rapid' *L.mono* agar were used to assess the injury of *Lm* after desiccation and sanitizer stress. We did not perform such experiments using 8000 ppm of quat because a much larger volume (i.e., at least 10 mL) of D/E broth was needed and it was impossible to rinse and recover *Lm* from each square with that large volume of liquid. We also performed quantitation of *Lm* cells after desiccation, exposure to sanitizer and sponge sampling. We tested two combinations, 800 CFU/square inoculation with 400 ppm of quat and 3000 CFU/square with 8000 ppm of quat, used sponges pre-moistened in D/E broth to swab each sampling square as described above, and then used 2 mL of D/E broth to rinse and recover any *Lm* remaining on each square. The 1.6 mL of collected D/E broth was plated onto 8 BHI agar plates.

### Statistical analysis

We used extended Fisher’s exact test in SAS v9.4 (Cary, NC) to compare the number of positive test portions out of the 20 test portions among the three neutralizing broths under each simulated sample storage scenario. When *p* value was less than 0.1, pairwise comparisons were performed.

We also performed chi-square analysis, which is prescribed in AOAC guidelines for two-way comparisons [26, 43], to compare between HiCap broth and D/E broth, and between Letheen broth and D/E broth.

## Data Availability

The dataset(s) supporting the conclusions of this article are included within the article.

## Abbreviations

BLEB: Buffered *Listeria* enrichment broth
D/E: Dey/Engley
AOAC International: Association of Official Analytical Collaboration International
HiCap: High capacity
QAC: Quaternary ammonium compound
RT: Room temperature
*Lm*: *Listeria monocytogenes*
BAM: Bacteriological Analytical Manual
MLG: Microbiology Laboratory Guidebook
NB: Neutralizing buffer
BPW: Buffered peptone water
BHI: Brain heart infusion

## References

[1] Kathariou S (2002). *Listeria monocytogenes* virulence and pathogenicity, a food safety perspective. J Food Prot.

[2] McCollum JT, Cronquist AB, Silk BJ, Jackson KA, O'Connor KA, Cosgrove S, Gossack JP, Parachini SS, Jain NS, Ettestad P (2013). Multistate outbreak of listeriosis associated with cantaloupe. N Engl J Med.

[3] Angelo KM, Conrad AR, Saupe A, Dragoo H, West N, Sorenson A, Barnes A, Doyle M, Beal J, Jackson KA, et al. Multistate outbreak of *Listeria monocytogenes* infections linked to whole apples used in commercially produced, prepackaged caramel apples: United States, 2014-2015. Epidemiol Infect. 2017;145(5):848–56. 10.1017/S0950268816003083 PMC6542465 28065170

[4] Chen Y, Luo Y, Pettengill J, Timme R, Melka D, Doyle M, Jackson A, Parish M, Hammack TS, Allard MW (2017). Singleton sequence type 382, an emerging clonal group of *Listeria monocytogenes* associated with three multistate outbreaks linked to contaminated stone fruit, caramel apples, and leafy green salad. J Clin Microbiol.

[5] Multistate outbreak of listeriosis linked to frozen vegetables (final update) [https://www.cdc.gov/Listeria/outbreaks/frozen-vegetables-05-16/]. Accessed 20 Oct 2020.

[6] Chen Y, Gonzalez-Escalona N, Hammack TS, Allard MW, Strain EA, Brown EW (2016). Core genome multilocus sequence typing for identification of globally distributed clonal groups and differentiation of outbreak strains of *Listeria monocytogenes*. Appl Environ Microbiol.

[7] Tan X, Chung T, Chen Y, Macarisin D, LaBorde L, Kovac J (2019). The occurrence of *Listeria monocytogenes* is associated with built environment microbiota in three tree fruit processing facilities. Microbiome.

[8] Ferreira V, Wiedmann M, Teixeira P, Stasiewicz MJ (2014). *Listeria monocytogenes* persistence in food-associated environments: epidemiology, strain characteristics, and implications for public health. J Food Prot.

[9] Gandhi M, Chikindas ML (2007). *Listeria*: a foodborne pathogen that knows how to survive. Int J Food Microbiol.

[10] Moretro T, Schirmer BCT, Heir E, Fagerlund A, Hjemli P, Langsrud S (2017). Tolerance to quaternary ammonium compound disinfectants may enhance growth of *Listeria monocytogenes* in the food industry. Int J Food Microbiol.

[11] Controlling cross contamination: optimal use of doorway sanitizers [https://www.qualityassurancemag.com/article/qa0213-doorway-sanitizers-use/]. Accessed 20 Oct 2020.

[12] Dry floor products won’t slip up [https://www.foodqualityandsafety.com/article/dry-floor-products-wont-slip-up/?singlepage=1]. Accessed 20 Oct 2020.

[13] Mohammad ZH, Hasan AA, Kerth CR, Riley DG, Taylor TM. Increased effectiveness of microbiological verification by concentration-dependent neutralization of sanitizers used in poultry slaughter and fabrication allowing *Salmonella enterica* survival. Foods. 2018;7(3):32. 10.3390/foods7030032 PMC5867547 29510486

[14] Park YJ, Chen J (2011). Mitigating the antimicrobial activities of selected organic acids and commercial sanitizers with various neutralizing agents. J Food Prot.

[15] Kamel A, Tomasino SF (2017). Analytical method for the detection of residual active ingredients found in neutralized suspensions of antimicrobial products. J AOAC Int.

[16] Gilbert SE, Rose LJ, Howard M, Bradley MD, Shah S, Silvestri E, Schaefer FW, Noble-Wang J (2014). Evaluation of swabs and transport media for the recovery of *Yersinia pestis*. J Microbiol Methods.

[17] Food and Drug Administration *Bacteriological Analytical Manual* Chapter 10, Detection and enumeration of *Listeria monocytogenes* in foods. http://www.fda.gov/Food/FoodScienceResearch/LaboratoryMethods/ucm071400.htm. Accessed 20 Oct 2020.

[18] An evaluation of HiCap™ neutralizing broth for detection of *Listeria* spp. from environmental sampling sponges dosed with three different types of sanitizers [https://iafp.confex.com/iafp/2014/webprogram/Paper6303.html]. Accessed 20 Oct 2020.

[19] Zhu L, Stewart D, Reineke K, Ravishankar S, Palumbo S, Cirigliano M, Tortorello M (2012). Comparison of swab transport media for recovery of *Listeria monocytogenes* from environmental samples. J Food Prot.

[20] Bazaco MC, Eifert JD, Williams RC, Kathariou S (2007). Quantitative recovery of *Listeria monocytogenes* and select salmonella serotypes from environmental sample media. J AOAC Int.

[21] Collection broths for environmental monitoring programs [https://www.fortrichard.com/uploads/resources/Environmental%20Monitoring/White%20Paper%20on%20HiCap%20Neutralizing%20Broth%20for%20Surface%20Sampling.pdf]. Accessed 20 Oct 2020.

[22] Environmental monitoring procedures [https://multimedia.3m.com/mws/media/241111O/environmental-monitoring-procedures-article.pdf]. Accessed 20 Oct 2020.

[23] Laird DT, Gambrel-Lenarz SA, Scher FM, Graham TE, Reddy R, Maturin LJ, Wehr HM, Frank JF (2004). Chapter 6. Microbiological count methods. Standard methods for the examination of dairy products.

[24] AOAC INTERNATIONAL Methods Committee Guidelines for Validation of Microbiological Methods for Food and Environmental Surfaces [http://www.aoac.org/aoac_prod_imis/AOAC_Docs/StandardsDevelopment/AOAC_Validation_Guidelines_for_Food_Microbiology-Prepub_version.pdf]. Accessed 20 Oct 2020.

[25] ISO: ISO 16140-2:2016 Microbiology of the food chain — Method validation — Part 2: Protocol for the validation of alternative (proprietary) methods against a reference method. In*.*; 2016.

[26] Sheth I, Li F, Hur M, Laasri A, Jesus AJD, Kwon HJ, Macarisin D, Hammack TS, Jinneman K, Chen Y (2018). Comparison of three enrichment schemes for the detection of low levels of desiccation-stressed *Listeria* spp. from select environmental surfaces. Food Control.

[27] Isolation and identification of *Listeria monocytogenes* from red meat, poultry and egg products, and environmental samples. Microbiology Laboratory Guidebook. http://www.fsis.usda.gov/wps/wcm/connect/1710bee8-76b9-4e6c-92fc-fdc290dbfa92/MLG-8.pdf?MOD=AJPERES [http://www.fsis.usda.gov/wps/wcm/connect/1710bee8-76b9-4e6c-92fc-fdc290dbfa92/MLG-8.pdf?MOD=AJPERES]. Accessed 20 Oct 2020.

[28] Sutton SV, Proud DW, Rachui S, Brannan DK (2002). Validation of microbial recovery from disinfectants. PDA J Pharm Sci Technol.

[29] Burnett SL, Beuchat LR (2002). Comparison of methods for fluorescent detection of viable, dead, and total *Escherichia coli* O157:H7 cells in suspensions and on apples using confocal scanning laser microscopy following treatment with sanitizers. Int J Food Microbiol.

[30] Laboratory Guidebook. Isolation and identification of *Listeria monocytogenes* from red meat, poultry and egg products, and environmental samples [http://www.fsis.usda.gov/wps/wcm/connect/1710bee8-76b9-4e6c-92fc-fdc290dbfa92/MLG-8.pdf?MOD=AJPERES]. Accessed 20 Oct 2020.

[31] Petrauskene OV, Cao Y, Zoder P, Wong LY, Balachandran P, Furtado MR, Tebbs RS (2012). Evaluation of applied biosystems MicroSEQ real-time PCR system for detection of *Listeria* spp. in food and environmental samples. J AOAC Int.

[32] Joelsson AC, Terkhorn SP, Brown AS, Puri A, Pascal BJ, Gaudioso ZE, Siciliano NA (2017). Comparative evaluation of Veriflow((R)) *Listeria* species to USDA culture-based method for the detection of *Listeria* spp. in food and environmental samples. J AOAC Int.

[33] Juck G, Gonzalez V, Allen AO, Sutzko M, Seward K, Muldoon MT (2018). Romer labs RapidChek((R)) *Listeria monocytogenes* test system for the detection of *L. monocytogenes* on selected foods and environmental surfaces. J AOAC Int.

[34] de Oliveira MM, Brugnera DF, Alves E, Piccoli RH (2010). Biofilm formation by *Listeria monocytogenes* on stainless steel surface and biotransfer potential. Braz J Microbiol.

[35] Lahou E, Uyttendaele M (2014). Evaluation of three swabbing devices for detection of *Listeria monocytogenes* on different types of food contact surfaces. Int J Environ Res Public Health.

[36] Parker A. Section IX, Effective cleaning and sanitizing procedures. In: Good Aquacultural Practices Manual: Joint Institute for Food Safety and Applied Nutrition. College Park: University of Maryland and FDA; 2007.

[37] Poimenidou SV, Chrysadakou M, Tzakoniati A, Bikouli VC, Nychas GJ, Skandamis PN (2016). Variability of *Listeria monocytogenes* strains in biofilm formation on stainless steel and polystyrene materials and resistance to peracetic acid and quaternary ammonium compounds. Int J Food Microbiol.

[38] Kragh ML, Truelstrup Hansen L (2019). Initial transcriptomic response and adaption of *Listeria monocytogenes* to desiccation on food grade stainless steel. Front Microbiol.

[39] Gruzdev N, Pinto R, Sela S (2011). Effect of desiccation on tolerance of *Salmonella enterica* to multiple stresses. Appl Environ Microbiol.

[40] Limoges M, Frontino G, Donnelly C. Comparative recovery of *Listeria* spp. From dairy environmental surfaces using 3M™ and World Bioproducts© environmental swabs with standard enrichment and enumeration methods. Food Control. 2020;114:107272.

[41] Letheen stability experiment [https://www.hygiena.com/index.php?option=com_docman&view=list&slug=tech-doc-sponge&Itemid=1134]. Accessed 20 Oct 2020.

[42] Zoz F, Grandvalet C, Lang E, Iaconelli C, Gervais P, Firmesse O, Guyot S, Beney L (2017). *Listeria monocytogenes* ability to survive desiccation: influence of serotype, origin, virulence, and genotype. Int J Food Microbiol.

[43] Alles S, Curry S, Almy D, Jagadeesan B, Rice J, Mozola M (2012). Reveal *Listeria* 2.0 test for detection of *Listeria* spp. in foods and environmental samples. J AOAC Int.

